# LIFE SATISFACTION AND SELF-PERCEPTION OF AGING IN CONTRIBUTING TO SUCCESSFUL AGING IN A CHINESE CONTEXT

**DOI:** 10.1093/geroni/igz038.531

**Published:** 2019-11-08

**Authors:** Sunny H W Chan, Yao Pan, Yuebin Xu, Ka Ching Yeung

**Affiliations:** 1 The Hong Kong Polytechnic University, Hong Kong, Hong Kong; 2 Beijing Normal University, Beijing, China; 3 The University of Hong Kong, Hong Kong, Hong Kong

## Abstract

Research indicated that life satisfaction and a positive self-perception of aging are vital during the aging process which contribute significantly to successful aging. Discovering the underlying determinants should provide a novel insight into the mechanism involved in achieving successful aging. A representative random sample of 2161 Chinese older people aged 60 years or above was surveyed by face-to-face interview. Sociodemographic factors were measured by age and educational level. Physical functioning was identified in terms of self-perceived health, basic and instrumental activities of daily living, and number of chronic illnesses. Social functioning was characterized in terms of number of people living together, social support network, and sense of loneliness. Hierarchical multiple linear regressions were performed to identify significant determinants of life satisfaction and self-perception of aging. Results showed that people in older age with lower educational level had a higher level of life satisfaction; whereas people in younger age with higher educational level had a more positive self-perception of aging. Moreover, social functioning took precedence over physical functioning in contributing to life satisfaction. In contrast, physical functioning outweighed social functioning in promoting a positive self-perception of aging. A sense of companionship and a supportive social network are imperative in enhancing life satisfaction, whereas perceived physical health and functional independence are essential in facilitating a positive self-perception of aging. This study provides empirical support to improve understanding of the primary mechanism of achieving successful aging. It also lays important groundwork for future tailored-made interventions for promoting successful aging in a Chinese context.

